# Dual-construct fixation is recommended in ipsilateral femoral neck fractures with infra-isthmus shaft fracture: A STROBE compliant study

**DOI:** 10.1097/MD.0000000000025708

**Published:** 2021-04-30

**Authors:** Yi Ping Wei, Kai Cheng Lin

**Affiliations:** Department of Orthopedic, Kaohsiung veteran general hospital, Kaohsiung, Taiwan.

**Keywords:** basicervical neck fracture, ipsilateral femoral neck and shaft fractures, malreduction

## Abstract

The aim of this study was to evaluate the risk factors related to osteosynthesis failure in patients with concomitant ipsilateral femoral neck and shaft fractures, including old age; smoking habit; comminuted fragments; infra-isthmus fracture; angular malreduction; unsatisfactory reduction (fracture gap >5 mm); and treatment with single construct.

Patients over the age of 20 with concomitant ipsilateral femoral neck and shaft fractures diagnosed at a level one medical center between 2003 and 2019 were included. Treatment modalities included single construct with/without an antirotational screw for the neck and dual constructs. Radiographic outcomes were assessed from anteroposterior and lateral hip radiographs at follow-up. Fisher exact test was used to analyze categorical variables. The presence of avascular necrosis of the femoral head, delayed union, atrophic or hypertrophic nonunion of the femoral shaft fracture, and loss of reduction were identified as factors related to treatment failure.

A total of 22 patients were included in this study. The average age was 58.5 years, and the majority was male (68.2%). The minimum radiographic follow-up duration was 12 months, and the median follow-up time was 12 (interquartile range 12–24) months.

Femoral neck osteosynthesis failed in 3 patients, whereas femoral shaft osteosynthesis failed in 12 patients. Fisher exact test demonstrated the failure of femoral shaft osteosynthesis was significantly more frequent in the single-construct cohort in 16 infra-isthmus femoral fracture cases (*P* = .034).

In ipsilateral femoral neck and infra-isthmus shaft fractures, it is better to treat the neck and shaft fractures with separate implants (dual constructs).

In a dual-construct cohort, separate plate fixation of the femoral shaft achieved a better result in terms of bone union than retrograde nailing of the shaft (bone union rate: 4/8 vs 0/2).

## 1 Introduction

Ipsilateral femoral neck and shaft fractures have been challenging for orthopedic surgeons since they were first described by Delaney and Street in 1953.^[1]^ To our knowledge, up to 9% of femoral shaft fractures have an associated femoral neck fracture, and up to 30% of femoral neck fractures associated with femoral shaft fractures are missed upon initial assessment.^[2]^

Regarding the morphological characteristics of these fractures, the shaft fracture is typically comminuted, in the middle shaft (52%–95%), and 15% to 33% of cases are open fractures.^[2]^ The neck fracture is usually basicervical, vertically oriented, and is nondisplaced in 60% of cases.^[3]^

In the past decade, much research has focused on treatment modalities. The options include: single construct with/without an antirotational screw for the neck (eg, cephalomedullary nail, reconstruction-type nail, or long dynamic hip screw)^[4–7]^ and dual constructs (a dynamic hip screw [DHS] or cancellous screws for the neck, with a separate plate or retrograde nailing for the shaft).^[6,8–11]^ More than 60 suggested options for treatment have been described in the literature, but no method has been proven to be exclusively more effective than the others.^[12]^ Low-level evidence from a case series suggested that separate implants may result in fewer reoperations.^[13]^ Some surgeons have recommended fixation with dual implants because the use of a single cephalomedullary device often results in more complications and malunion.^[3]^

The study aimed to evaluate and identify risk factors resulting in osteosynthesis failure in patients with ipsilateral femoral neck and shaft fractures. Our parameters were selected based on current literature and the clinical experience of the senior author.^[1,2,3,4,7,13,14]^

## 2 Methods

After receiving the research ethics board's approval (KSVGH20-CT3-12), we retrospectively evaluated patients between 2003 and 2019 from a single hospital. The inclusion criteria for the study covered all adults with concomitant ipsilateral femoral neck and shaft fractures treated in our level-one trauma center during the study period. The femoral neck fractures were categorized via subcapital, transcervical, or basicervical region; the femoral shaft fractures were classified as AO-Müller/Orthopedic Trauma Association (AO/OTA) long-bone fracture classification. Patients with intertrochanteric fractures, subtrochanteric fractures, distal femoral fractures (AO/OTA-33), iatrogenic femoral neck fractures, and neglected femoral neck fractures were all excluded.

In this study, we evaluated the radiography and medical records, which are performed by an independent orthopedic surgeon who was not involved in the surgery. The evaluation consisted of regular radiographic images of the hip, such as anteroposterior and lateral at the initial of trauma to evaluate the pattern. Otherwise, medical records, including age, sex, and other characteristics, were reviewed for each patient.

With the lack of consensus about the optimal fixation technique, this cohort study aimed to evaluate the postoperative outcomes of the dual fractures comparing single and seperate implant fixation.

### 2.1 Protocols of postoperative treatment

Out protocol of postoperative treatment is non–weight-bearing ambulation for 3 months. Gradual passive range of motion to active range of motion of hip and knee joint is started after surgery. The partial weight-bearing ambulation with a walker could begin since 3 months postoperatively once no malalignment was noted in the radiographic examination. After surgery, radiographic imaging will be followed up once every 3 months until the patients had clinical and radiographic healing, and the outpatient follow-up will maintain at least for 1 year.

### 2.2 Main outcome measures

During follow-up, we examined anteroposterior and lateral hip radiographs to assess the quality of the reduction, secondary displacement, consolidation, and the presence of avascular necrosis changes in the hip joint at 6-month and 9-month follow-up. The subsequent avascular necrosis of the femoral head was evaluated and classified according to the Ficat classification. Fracture malreduction was defined as an angulation >5 degrees in any plane or shortening of >1 cm. Fractures were considered union when radiographs showed 3 bone bridge cortices combined with no pain/tenderness at the fracture site.^[15]^ Delayed union was considered if the time exceeded 6 months after the primary operation. Nonunion was considered if no bone consolidation was seen 9 months after the injury.^[15]^ The presence of avascular necrosis of the femoral head, delayed union, atrophic or hypertrophic nonunion of the femoral shaft fracture, and loss of reduction all indicated treatment failure.

### 2.3 Statistics

Data were processed and analyzed using the Statistical Package for the Social Sciences (SPSS 20.0, SPSS Inc, Chicago, IL). The categorical variables were analyzed by nonparametric statistical analysis with Fisher exact test because of the small sample size. The difference was statistically significant when *P* < .05. We used Pauwel angle of 50 degrees as cut-off based on Pauwel classification to stratify the study cohort for analysis (Pauwel >50 vs Pauwel ≤50).^[2,4,13]^

## 3 Results

One patient with ipsilateral distal femoral fractures was excluded. A total of 22 patients were included in this study (Table 1). There were no significant differences in treatment decisions related to demographic factors such as age, sex, or fracture pattern (wedge, segmental, or comminuted fragment) (Table 2). The mean (±standard deviation) age at the time of fracture was 45.18 (±16.00) years. There were 15 males and 7 females; 12 left leg fractures and 10 right leg fractures; and 7 patients suffered major trauma (Injury Severity Score >16). The minimum radiographic follow-up duration was 12 months, and the median follow-up time was 12 (interquartile range, 12–24) months. Historically, the overall nonunion rate of femoral neck and shaft fractures in this combined injury pattern is 5% and 20%, respectively. However, in our 17-year experience at our hospital, we found a much higher nonunion rate of the femoral shaft in this combined injury pattern than has previously been reported.^[14]^

**Table 1:** Characteristics of the 22 patients.

Age, y	Sex	Smoking habit	Pauwel angle of neck fracture	AO/OTA classification 32 (shaft)	Isthmus or infraisthmus fracture	Type of treatment (1 or 2 constructs)	Angular malreduction of shaft	Failure of neck osteosynthesis	Failure of shaft osteosynthesis
46	M	+	47	A3	Infra-	2 Constructs (separate plate)	—		
38	M	+	56	C2	Infra-	1 Construct (CM nail^∗^ with an antirotational screw)	—		Failure (hypertrophic)
47	F	—	66	A2	Infra-	1 Construct (RC nail^†^ with an antirotational screw)	—		Failure (hypertrophic)
34	F	—	48	A3	Infra-	2 Constructs (separate plate)	—		
33	M	—	59	A3	Isthmus	1 Construct (long DHS with an antirotational screw)	+		
77	M	—	81	A3	Infra-	2 Constructs (separate plate)	—	Failure (loss of reduction)	
43	F	—	56	B2	Infra-	2 Constructs (separate plate)	—		
68	M	—	66	B2	Infra-	1 Construct (CM nail)	—		Failure (hypertrophic)
58	F	—	51	A3	Isthmus	1 Construct (CM nail)	+	Failure (loss of reduction)	Failure (loss of reduction)
20	F	—	48	B2	Isthmus	1 Construct (CM nail)	—		
55	F	—	31	A3	Infra-	2 Constructs (separate plate)	—	X^‡^	
30	M	—	64	B2	Isthmus	1 Construct (CM nail)	—		
80	M	+	70	B2	Infra-	2 Constructs (separate plate)	—		Failure (broken plate)
36	M	—	57	B2	Infra-	1 Construct (RC nail with an antirotational screw)	—		Failure (hypertrophic)
48	M	+	58	C1	Infra-	2 Constructs (retrograde nail)	—		Failure (hypertrophic)
32	M	+	69	A3	Infra-	2 Constructs (retrograde nail)	—		Failure (atrophic)
50	F	—	56	C2	Infra-	1 Construct (CM nail)	+		Failure (hypertrophic)
34	M	+	60	A2	Isthmus	1 Construct (RC nail)	—		
51	M	—	61	A1	Infra-	1 Construct (RC nail with an antirotational screw)	—		Failure (hypertrophic)
25	M	—	78	A3	Infra-	2 Constructs (separate plate)	—		Failure (hypertrophic)
57	M	—	56	A3	Infra-	1 Construct (antegrade nail with antirotational pins)	—		Failure (hypertrophic)
32	M	—	48	A3	Isthmus	2 Constructs (separate plate)	—	Failure (avascular necrosis)	

**Table 2:** Patient demographics.

Risk factors	Treatment with a single construct	Treatment with double constructs	Fisher *P*^∗^
Old age (> 60 y)	4	4	1
Sex (male)	8	7	1
Smoking habit	2	4	.348
Major trauma (Injury Severity Score ≥16)	3	4	.652
Basicervical fracture	4	3	1
Pauwel angle >50 degrees	11	6	.135
Wedge, segmental, or comminuted fragment (AO/OTA classification B and C)	6	3	.415
Infra-isthmus fracture	7	9	.162

The timing of operation was often dictated by the patient's status as a multiple trauma victim. Delay in treatment was generally because of the associated injuries (head, chest, or abdominal). The rate of avascular necrosis of the femoral head was 4%, and a delay of 5 to 6 days in fixation of the neck fracture did not seem to increase this complication rate in the present study.^[16–18]^ All our 22 patients were operated in 24 to 72 hours after trauma.

Although there is confusion regarding which fracture (femoral neck or shaft) should be managed first, there appears to be a general consensus in our hospital regarding the seriousness of the complications involving femoral neck fractures. In medical records, stabilizing femoral neck fractures at first in 21 patients was detected. (One patient received plate fixation for the shaft and then bipolar hemiarthroplasty for the neck.)

### 3.1 Fracture pattern of the femoral shaft

The radiographic AO/OTA-32 classifications of our patients were as follows: 13 type A fractures (1 A1, 2 A2, 10 A3), 6 type B fractures (6 B2), and 3 type C fractures (1 C1, 2 C2). There was 1 open fracture, which was classified as Gustilo classification 2. A total of 12 patients were treated with a single construct, whereas 10 patients were treated with double constructs. There were no statistically significant differences between fracture patterns (AO/OTA classification-A type compared with B and C types) with regards to treatment modality (single construct or dual constructs) according to Fisher test (*P* = .415) (Table 2). Sixteen shaft fracture sites were infra-isthmus femoral fractures, and 6 were isthmus femoral fractures. We found statistically nonsignificant differences in treatment failure between fracture sites (isthmus or infra-isthmus) (*P* = .056) (Table 3). We therefore performed subgroup analysis of the 16 patients diagnosed with infra-isthmus femoral shaft fractures. Further analysis was performed to assess the impacts of risk factors on treatment failure in these 16 patients.

**Table 3:** Summary of clinical and radiographic findings of femoral shaft fracture by frequency, percentage, and Fisher exact test.

Risk factors	Risk factors present (% of 22 patients)	Failure of femoral shaft osteosynthesis (% of risk factors)	Fisher *P*^∗^
Nonmodifiable			
Open fracture	1 (4.5%)	1 (100%)	1
Smoking habit	6 (27.3%)	4 (66.7%)	.646
Major trauma (Injury Severity Score *≥*16)	7 (31.8%)	3 (42.9%)	.652
Wedge, segmental, or comminuted fragment (AO/OTA classification B and C)	9 (40.9%)	4 (44.4%)	.666
Infra-isthmus fracture	16 (72.7%)	11 (78.6%)	.056
Modifiable			
Angular malreduction of shaft	3 (13.6%)	2 (66.7%)	1
Separation gap >5 mm after osteosynthesis	1 (4.5%)	0	.455
Treatment with a single construct	12 (54.5%)	8 (66.7%)	.391
Treatment with double constructs	10 (45.5%)	6 (60.0%)	.691
Treatment with double constructs (separate plate for femoral shaft)	8 (36.4%)	4 (50.0%)	1
Treatment with double constructs (retrograde nail for femoral shaft)	2 (9.1%)	2 (100%)	.481

As shown in Table 4, subgroup assessment of risk factors in the 16 patients with infra-isthmus femoral shaft fractures was performed using Fisher exact test, and the results demonstrated that failure of femoral shaft osteosynthesis was significantly more frequent in the presence of an infra-isthmus femoral fracture treated with a single construct (*P* = .034).

**Table 4:** Assessment of risk factors in 16 patients with infra-isthmus femoral shaft fracture by frequency, percentage, and Fisher exact test.

Risk factors	Risk factors present (% of 16 patients)	Failure of femoral shaft osteosynthesis (% of risk factors)	Fisher *P*^∗^
Nonmodifiable			
Open fracture	1 (6.3%)	1 (100%)	1
Smoking habit	5 (31.3%)	4 (66.7%)	1
Wedge, segmental, or comminuted fragment (AO/OTA classification B and C)	7 (43.8%)	6 (85.7%)	.308
Modifiable			
Angular malreduction of shaft	1 (6.3%)	1 (100%)	1
Separation gap >5 mm after osteosynthesis	0	0	1
Treatment with a single construct	7 (43.8%)	7 (100%)	.034
Treatment with double constructs	9 (56.3%)	4 (57.1%)	.034
Treatment with double constructs (separate plate for femoral shaft)	7 (43.8%)	2 (28.6%)	.005
Treatment with double constructs (retrograde nail for femoral shaft)	2 (12.5%)	2 (100%)	1

Among all predictors, no one was independently associated with infraisthmus fracture site (Table 4). In infraisthmus fracture cohort, 7 patients received the treatment with single construct, and all 7 patients developed treatment failure in shaft region. Five in 7 patients exhibited bone union of the shaft after operation with double-construct with separate plate fixation. However, due to small sample size, we could not prove statistical significance between fracture site (infraisthmus) and treatment (double-construct with separate plate) in regression analysis.

### 3.2 Fracture pattern of the neck

Fisher exact test was performed to assess antirotational screw use, the results of which are summarized in Table 5. Compared to treatment without an antirotational device (pin or screw), treatment with antirotational device fixation of the neck did not produce uniformly more favorable results (*P* *=* .526).

**Table 5:** Summary of clinical and radiographic findings of femoral neck fracture by frequency, percentage, and Fisher exact test.

Risk factors	Risk factors present (% of 21 patients^∗^)	Failure of femoral neck osteosynthesis (% of risk factors)	Fisher *P*^†^
Nonmodifiable			
Old age (>60 y)	3 (14.3%)	0	1
Smoking habit	6 (28.6%)	0	.526
Major trauma (Injury Severity Score *≥*16*)*	6 (28.6%)	1 (16.7%)	1
Basicervical fracture	7 (33.3%)	1 (14.3%)	1
Pauwel angle > 50 degrees	17 (81.0%)	2 (11.8%)	.489
Displaced neck fracture (Garden type 3 or 4)	11 (52.4%)	2 (18.2%)	1
Modifiable			
Malreduction of neck	1 (4.8%)	1 (100%)	.143
Treatment with a single construct (with an antirotational device)	6 (28.6%)	0	.526
Treatment with a single construct (without an antirotational device)	6 (28.6%)	1 (16.7%)	1
Treatment with double constructs	9 (42.9%)	2 (22.2%)	.553

### 3.3 Outcome measures at follow-up

Primary anatomical reduction was achieved in 22 (100%) cases; however, at the end of the follow-up period, there were only 8 (36.4%) excellent and good results. Two patients had loss of reduction (in 2 neck fractures and 1 shaft fracture); 1 patient sustained a broken plate; 9 patients exhibited hypertrophic nonunion of the shaft; 1 patient experienced atrophic nonunion of the shaft; and 1 patient suffered avascular necrosis of the femoral head (Ficat stage IV). The revision surgeries and their outcomes are all listed in Table 6.

**Table 6:** Revision surgeries in cases of failed primary osteosynthesis and their outcomes^∗^.

Primary surgery	Duration between primary surgery and revision surgery	Revision surgery	Outcome of revision surgery
Case with dual constructs (DCP and cannulated screws^∗^3)	6 mo; loss of reduction of neck	Bipolar hemiarthroplasty	
Case with single construct (CM nail)	1 mo; loss of reduction of neck and shaft	Change to double constructs with retrograde nail for shaft and DHS for neck	Bone union
Case with dual constructs (LCP and cannulated screws^∗^3)	1 y; broken plate of shaft	Change to single construct with reconstruction nail	Bone union
Case with dual constructs (RC nail and cannulated screws^∗^3)	9 mo	Addition of autologous bone graft and side plate augmentation	Bone union
Case with single construct (CM nail)	1 y	Change to double constructs with strut fibular bone graft, DCP for shaft, and screws for neck	Bone union
Case with double constructs (DCP and cannulated screws^∗^3)	1 y; AVN change of femoral head	Bipolar hemiarthroplasty	

## 4 Discussion

Until now, none of the literature describes risk factors of treatment failure on femoral shaft in patients with concomitant ipsilateral dual fractures.

According to our analysis focusing on ipsilateral femoral neck and shaft fracture, applying separate implants for internal fixation is recommended. Furthermore, the major issue we experienced was related to the different fracture sites (supra-isthmus, isthmus, or infra-isthmus femoral shaft), as the site influences the choice of treatment and surgical technique.

There is still no consensus regarding the optimal treatment method for these complex fractures. However, most surgeons agree that reduction and fixation of a femoral neck should take priority to preserve the head.

Retrograde nailing for femoral shaft fractures, with separate fixation for the femoral neck fracture, was suggested by Oh et al.^[11]^ Easy fixation and favorable results have been reported. There were 16 good and 1 fair functional results; 1 patient with a fair result underwent total hip arthroplasty due to nonunion of the femoral neck with avascular necrosis. Theoretically, this seems to be an attractive treatment modality. Ostrum et al^[19]^ reported a multicenter series of 95 patients with dual fractures who underwent retrograde nailing and fixation with either cannulated screws or a DHS. The authors reported union rates of 98% for the femoral neck fractures and 91.3% for the femoral shaft fractures. Comminution and initial displacement of the proximal femoral fracture may lead to malunion or nonunion.

However, analysis of 2 series demonstrated a lower diaphyseal union rate with retrograde femoral nails.^[5,11]^ Recently, a multicenter retrospective review of 89 patients suffered from this dual fractures and treated with separate neck fixation and a retrograde nail.^[20]^ Higher nonunion rate of femoral shaft was seen in retrograde nail group than in antegrade nail group. (80% vs 20%)

Both of our retrograde femoral nails were placed after reaming. In our study, both cases treated with retrograde nails were femoral neck and infra-isthmus shaft fractures. One case was an open fracture (Gustilo type 2), and hypertrophic nonunion was diagnosed in the femoral shaft region at follow-up. The other patient was a heavy smoker, and sustained atrophic nonunion of the femoral shaft fracture. Ipsilateral femoral neck and shaft fractures can be seen as a long segmental femoral fracture. Owing to the “long floating middle fragment” of these dual fractures, it is difficult to achieve stability when using small-diameter retrograde nails in the infraisthmus region. The absence of stability may progress to hypertrophic nonunion despite an initial perfect reduction. Furthermore, the working length in our 2 cases was shorter than in other cases fixed with antegrade nails because the proximal femur had to be preserved for the separate fixation of the femoral neck fracture. The shorter working length may influence nail stiffness under bending and torsion.

A case series,^[21]^ published in 2020, demonstrate an interesting management strategy to increase the stability when treating with retrograde nail in these dual fractures. The “rendezvous technique" using dual implants in an overlapping fashion to achieve the longer working length and more stabilities.

**Figure 1:**
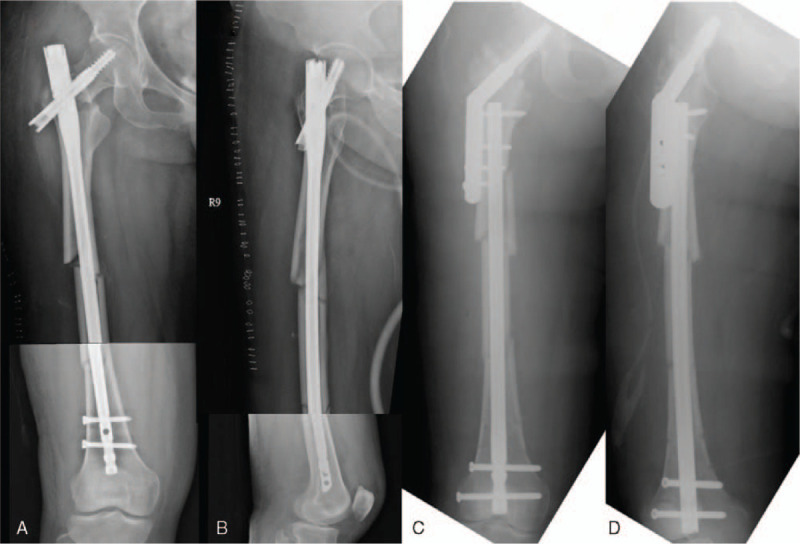
(A and B) Post-op 1-month plain view. Ipsilateral right femoral middle shaft fracture AO 32-A3 and Garden type IV, Pauwel angle: 51-degree femoral neck fracture. Treatment with CM nail with loss of reduction. (C and D) Post-revision surgery plain view. Treatment with dual implants by rendezvous technique (DHS and retrograde nail).

**Figure 2:**
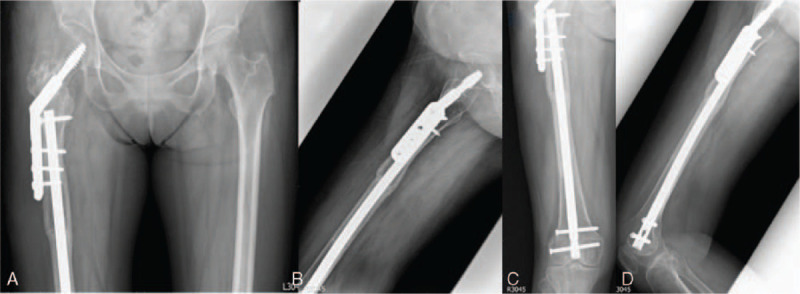
(A–D) Post-op 1-year follow-up. Consolidation was achieved both at femoral neck and femoral shaft region.

There are some benefits of the “rendezvous” technique, particularly in a low resource setting which is lack of assistant in operation room. One of our cases with initial loss of reduction received the revision surgery with retrograde nail and DHS by the “rendezvous” technique. And bone union was seen at postoperative 9 months and good clinical outcome (range of motion of knee joint: 0–120 degree, flexion) was also reported (Figs. 1 and 2 and Table 6.)

Plate fixation of the femoral shaft with separate fixation of the femoral neck has been widely used in the past.^[22,23]^ The advantages of which include reliable and familiar methods of fixation for each fracture, and intraoperative compression can be obtained by tightening screws down a ramped hole design on the plate. This method effectively decreases the likelihood of both malalignment and shortening. The disadvantages include increasing blood loss and periosteal stripping of the femoral shaft, extensive surgical dissection, and the potential need for a bone graft.

With our dual constructs and separate plating group (N = 8), we experienced a nonsignificant difference in the treatment failure rates of femoral neck and shaft osteosynthesis (failure rate: 4/8 in shaft fractures, and 2/8 in neck fractures). In our 16 cases of “infraisthmus” femoral shaft fracture, dual constructs with separate plate fixation appeared to be a reliable method by which to lower the failure rate (failure rate: 2/7 cases; *P* = .005). In our case series, 6 patients received dynamic compression plate fixation, while only 1 patient received locking compression plate fixation, and a broken locking plate was noted at the 1-year follow-up.

Nirmal et al^[24]^ reported a prospective analysis of the two methods (single or double-construct) in 18 cases. Dual implants gave better functional results even in patients with a displaced neck fracture. Tsai et al^[25]^ performed a retrospective study of 43 cases treated with single construct, and owing to a high complication rate for the femoral neck region; single-construct treatment with antegrade nail and screw fixation was not recommended for these dual fractures. Conclusions of a study of a larger series from the same institution stated: “fixation schemes that rely on one device for both fractures seem to compromise the treatment of one or both fractures in some way.”^[26–28]^

We experienced a high nonunion rate in our patients with ipsilateral femoral neck and “infraisthmus” shaft fractures who underwent antegrade nailing (7/7; 100%). The failure rate was higher for the femoral shaft region than the femoral neck region (11/16 cases; 1/16 cases).

Literature reported the nonunion rate is higher for “infraisthmus” femoral shaft fractures as compared with isthmus fractures.^[29]^ In ipsilateral femoral neck and shaft fractures, the free and floating segment between the two fracture sites may create more motion and instability than would be encountered at an isolated femoral fracture site, especially in dual fractures with an “infraisthmus” shaft fracture.^[30]^ The larger medullary cavity of infraisthmus fractures, causes the “pendulum phenomenon,” and increases the likelihood of ipsilateral injuries progressing to nonunion.^[29]^ In other words, when an antegrade nail is employed for treatment, the nail can stabilize the proximal and the isthmus, but the distal femoral medullary cavity is larger, and 2 or 3 distal locking screws cannot limit the nail, which decreases the stability of the construct. It is difficult to achieve absolute stability in a large distal femoral medullary cavity with nailing using 2 to 3 distal screws. Furthermore, different fracture sites should be analyzed separately, as the site influences the choice of treatment and surgical technique. Hence, the fracture site (supra-isthmus, isthmus, or infra-isthmus) was taken into consideration when comparing outcomes in our study. To our best of knowledge, none of literature reported the risk of nonunion in different anatomical fracture sites in these dual fractures. This forms our new contribution to the current literature.

In our institute, most patients (16/22; 72.7%) with dual fractures had infra-isthmus femoral shaft fractures; 11 of the 16 patients (78.6%) were diagnosed with nonunion of the shaft fracture (*P* = .056) (Table 3), and this may be the main reason for the higher failure rate of femoral shaft osteosynthesis in these dual fractures in our study than previously reported.

Limitations of this study included the relatively small size of the cohort, the variety of implants used, surgical options based on different surgeons, and the retrospective design.

## 5 Conclusions

In ipsilateral femoral neck and “infraisthmus” shaft fractures, it is better to treat the neck and shaft fractures with separate implants (dual constructs).

In the dual constructs cohort, separate plate fixation of the femoral shaft achieved a better result in terms of bone union than retrograde nailing of the shaft (bone union rate: 4/8 vs 0/2).

## Author contributions

**Conceptualization:** Yi Ping Wei, Kai Cheng Lin.

**Data curation:** Yi Ping Wei, Kai Cheng Lin.

**Supervision:** Kai Cheng Lin.

**Writing – original draft:** Yi Ping Wei.

**Writing – review & editing:** Kai Cheng Lin.
